# The monoclinic form of di-μ-aqua-bis­[diaqua­bis­(thio­cyanato-κ*N*)iron(II)]–1,4-bis­(4*H*-1,2,4-triazol-4-yl)benzene (1/3)

**DOI:** 10.1107/S160053681202613X

**Published:** 2012-07-10

**Authors:** Yuan-Yuan Liu, Pan Yang, Bin Ding

**Affiliations:** a Tianjin Key Laboratory of Structure and Performance for Functional Molecule, Tianjin Normal University, Tianjin 300071, People’s Republic of China

## Abstract

The title complex, [Fe_2_(NCS)_4_(H_2_O)_6_]·3C_10_H_8_N_6_, comprises two Fe^II^ atoms octahedrally coordinated and bridged by two aqua O atoms that straddle a crystallographic inversion center, forming a quadrilateral core. The water ligands of the core are involved in hydrogen bonds with the triazole N atoms of the organic mol­ecules, which generates a layer motif in the *ab* plane. There are π–π stacking inter­actions between benzene rings of 3.490 (6) Å, and between triazole rings of 3.543 (8) and 3.734 (7) Å in neighboring layers, forming a three-dimensional network.

## Related literature

For details of compounds containing similar diiron centers, see: Hsu *et al.* (1999 ▶); Zheng *et al.* (1999 ▶); MacMurdo *et al.* (2000 ▶); Yoon *et al.* (2004 ▶). For information on multicomponent di­oxy­gen dependent enzymes including toluene monooxygenase, see: Sazinsky *et al.* (2004 ▶), and for those that include the *R*_2_ subunit of ribonucleotide reductase, see: Nordlund & Eklund (1993 ▶); Stubbe & Van der Donk (1998 ▶). For the triclinic form of the title compound, see: Yang *et al.* (2012 ▶).
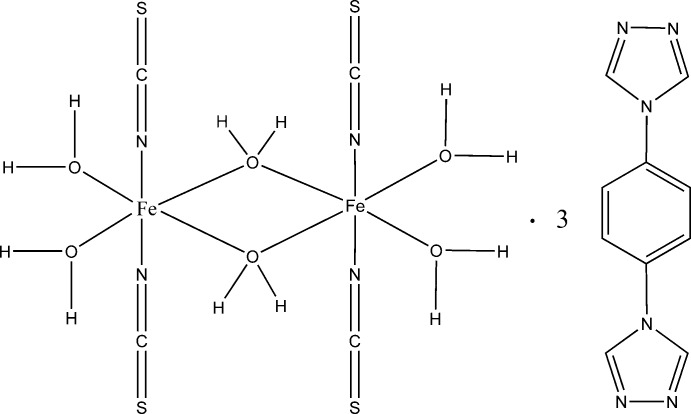


## Experimental

### 

#### Crystal data

[Fe_2_(NCS)_4_(H_2_O)_6_]·3C_10_H_8_N_6_
*M* *_r_* = 1088.79
Monoclinic, 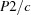

*a* = 7.828 (2) Å
*b* = 14.198 (4) Å
*c* = 19.846 (5) Å
β = 97.212 (4)°
*V* = 2188.3 (10) Å^3^
*Z* = 2
Mo *K*α radiation
μ = 0.93 mm^−1^
*T* = 173 K
0.18 × 0.17 × 0.16 mm

#### Data collection

Bruker APEXII CCD diffractometer
Absorption correction: multi-scan (*SADABS*; Sheldrick, 1996 ▶) *T*_min_ = 0.851, *T*_max_ = 0.866
10801 measured reflections
3865 independent reflections
2796 reflections with *I* > 2σ(*I*)
*R*_int_ = 0.058

#### Refinement

*R*[*F* ^2^ > 2σ(*F* ^2^)] = 0.050
*wR*(*F* ^2^) = 0.132
*S* = 1.04
3865 reflections
309 parameters
1 restraint
H-atom parameters constrained
Δρ_max_ = 0.89 e Å^−3^
Δρ_min_ = −0.56 e Å^−3^

### 

Data collection: *APEX2* (Bruker, 2007 ▶); cell refinement: *SAINT* (Bruker, 2007 ▶); data reduction: *SAINT*; program(s) used to solve structure: *SHELXS97* (Sheldrick, 2008 ▶); program(s) used to refine structure: *SHELXL97* (Sheldrick, 2008 ▶); molecular graphics: *SHELXTL* (Sheldrick, 2008 ▶) and *DIAMOND* (Brandenburg, 1999 ▶); software used to prepare material for publication: *publCIF* (Westrip, 2010 ▶).

## Supplementary Material

Crystal structure: contains datablock(s) global, I. DOI: 10.1107/S160053681202613X/pk2413sup1.cif

Structure factors: contains datablock(s) I. DOI: 10.1107/S160053681202613X/pk2413Isup2.hkl

Additional supplementary materials:  crystallographic information; 3D view; checkCIF report

## Figures and Tables

**Table 1:** Selected bond lengths (Å)

Fe1—N12	2.080 (3)
Fe1—N11	2.098 (3)
Fe1—O1	2.099 (2)
Fe1—O3	2.106 (2)
Fe1—O2	2.258 (2)
Fe1—O2^i^	2.271 (2)

Symmetry code: (i) 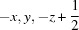.

**Table 2:** Hydrogen-bond geometry (Å, °)

*D*—H⋯*A*	*D*—H	H⋯*A*	*D*⋯*A*	*D*—H⋯*A*
O1—H1*A*⋯N3^ii^	0.84	2.00	2.833 (4)	176
O1—H1*B*⋯N6^iii^	0.84	2.00	2.841 (4)	178
O2—H2*A*⋯N10	0.84	2.00	2.834 (3)	176
O2—H2*B*⋯N8^iv^	0.84	2.00	2.836 (4)	174
O3—H3*A*⋯N2^i^	0.84	1.95	2.784 (4)	176
O3—H3*B*⋯N5^v^	0.84	1.92	2.761 (4)	179

Symmetry codes: (i) 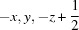; (ii) 
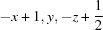
; (iii) 
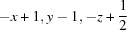
; (iv) 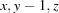; (v) 
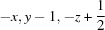
.

**Fig. 1.**
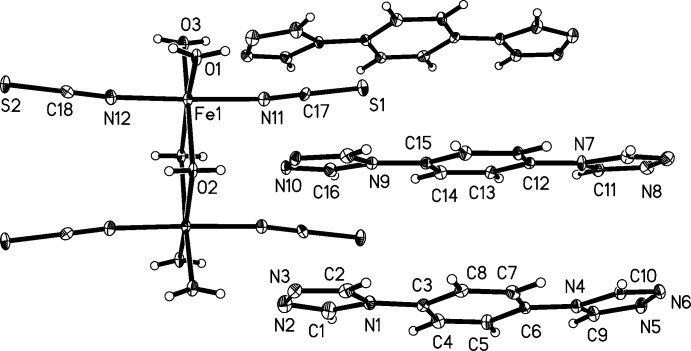
The structure of the title complex, showing 30% probability displacement ellipsoids. Unlabelled atoms of the inversion-related parts have symmetry code: (-x, y, -z + 1/2).

**Fig. 2.**
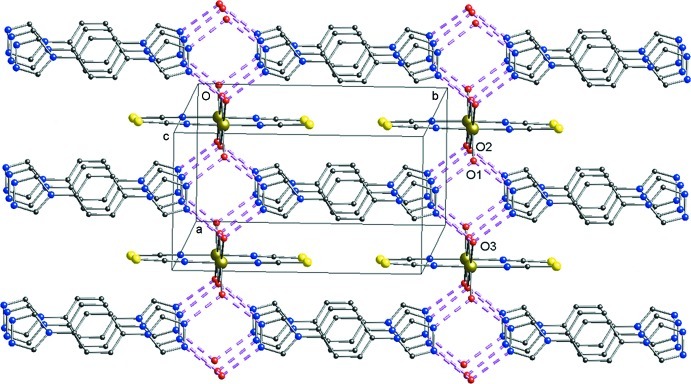
The two-dimensional layer structure of the title complex viewed approximately along the crystallographic c axis. Purple dashed lines indicate O—H···N hydrogen bonds.

## supplementary crystallographic information

### Comment

The diiron unit, with a carboxylate-rich coordination environment, continues to attract considerable attention due to its role in enzyme catalysis activity, which occurs in related multicomponent dioxygen dependent enzymes, including toluene monooxygenase (Sazinsky *et al.*, 2004), the *R*_2_ subunit of ribonucleotide reductase (Stubbe & Van der Donk, 1998; Nordlund & Eklund, 1993). With the development of compounds that contain the diiron center, the structure of a series of Fe2(II,II) (MacMurdo *et al.*, 2000), Fe2(III,III) (Zheng *et al.*, 1999) and Fe2(III,IV) (Hsu *et al.*, 1999) complexes with a central Fe_2_O_2_ quadrilateral have been obtained. In comparison to other compounds with similar di-iron species, it is rare for the quadrilateral center to include two water molecules, as most contain carboxylic oxygen atoms. In order to explore further details of the coordination environment of the diiron system, the title complex was synthesized. As shown in Fig. 1, the structure comprises two distorted octahedral iron(II) centers that straddle a crystallographic inversion center bridged by two aqueous oxygen atoms to form a quadrilateral core. The separation between the iron atoms is 3.498 (4) Å, which is remarkably different from the 3.0430 (7) Å reported previously, owing to the absence of carboxylate ligands (Yoon *et al.*, 2004). Moreover, the distance of Fe—Fe is different from that in complexes that contain higher valence iron (MacMurdo *et al.*, 2000; Zheng *et al.*, 1999; Hsu *et al.*, 1999). The bond lengths of Fe—O2 and Fe—O2A are 2.258 (3) and 2.271 (5) Å, and the angles of O2—Fe—O3 and Fe1A—O2—Fe are 92.09 (1)° and 101.12 (9)°. Each Fe(II) center resides in a six-coordinated octahedron of two nitrogens and four waters. Two waters bridge the iron atoms in the equatorial plane to form the quadrilateral core with a mean Fe-O distance of 2.272 (2) Å. The other waters (O1 & O3) act as terminal ligands with the bond lengths 2.106 (4) Å and 2.099 (4) Å. The axial positions are occupied by two N atoms from the NCS^-^ anions with the distances 2.080 (1) Å and 2.098 (1) Å to iron. Selected bond distances are listed in Table 1. As indicated in Fig. 2, classical intermolecular O—H···N H-bonds are formed between the triazole nitrogen atom supplied by the uncoordinated organic ligand 1,4-Bis(4*H*-1,2,4-triazol-4-yl)benzene and aqueous oxygen atoms supplied by the bridging and terminal water ligands to generate a two-dimensional ladder-like structure with the O···N separation ranging from 2.761 (4) Å to 2.861 (4) Å. In addition, there are intermolecular π-π stacking interactions between the organic species within the crystal that form a three-dimensional network. The interlayer distance between triazole moieties and benzene rings of neighboring layers is 3.490 (6) Å. There are also π-π stacking interactions between the triazole moieties within one layer, the corresponding distance are 3.543 (8) Å and 3.734 (7) Å. Details of the hydrogen bonds are given in Table 2.

### Experimental

The compound was synthesized under hydrothermal conditions. A mixture of *L* (*L* = 1,4-bis(4*H*-1,2,4-triazol-4-yl)benzene) (0.3 mmol, 0.0636 g), FeSO_4_.7H_2_O (0.1 mmol, 0.028 g), KSCN (0.2 mmol, 0.019 g) and water (10 ml) was placed in a 25 ml acid digestion bomb and heated at 433 K for two days, then slowly cooled to room temperature over three days. After washing twice with 5 ml water, colorless block crystals of the compound were obtained.

### Refinement

The water H atoms were located in a Fourier difference map and refined subject to an O—H distance restraint of 0.88 (1) Å and an H···H distance restraint of 1.42 (2) Å. Other H atoms were allowed to ride on their parent atoms with C—H distances of 0.93 Å (*U*_iso_(H) = 1.2Ueq(C)). All of the non-hydrogen atoms were refined anisotropically.

### Crystal data

**Table:**

[Fe_2_(NCS)_4_(H_2_O)_6_]·3C_10_H_8_N_6_	*F*(000) = 1116
*M**_r_* = 1088.79	*D*_x_ = 1.652 Mg m^−^^3^
Monoclinic, *P*2/*c*	Mo *K*α radiation, λ = 0.71073 Å
Hall symbol: -P 2yc	Cell parameters from 2057 reflections
*a* = 7.828 (2) Å	θ = 2.5–27.8°
*b* = 14.198 (4) Å	µ = 0.93 mm^−^^1^
*c* = 19.846 (5) Å	*T* = 173 K
β = 97.212 (4)°	Block, colourless
*V* = 2188.3 (10) Å^3^	0.18 × 0.17 × 0.16 mm
*Z* = 2	

### Data collection

**Table:**

Bruker APEXII CCD diffractometer	3865 independent reflections
Radiation source: fine-focus sealed tube	2796 reflections with *I* > 2σ(*I*)
Graphite monochromator	*R*_int_ = 0.058
φ and ω scans	θ_max_ = 25.0°, θ_min_ = 1.4°
Absorption correction: multi-scan (*SADABS*; Sheldrick, 1996)	*h* = −8→9
*T*_min_ = 0.851, *T*_max_ = 0.866	*k* = −16→16
10801 measured reflections	*l* = −21→23

### Refinement

**Table:**

Refinement on *F*^2^	1 restraint
Least-squares matrix: full	H-atom parameters constrained
*R*[*F*^2^ > 2σ(*F*^2^)] = 0.050	*w* = 1/[σ^2^(*F*_o_^2^) + (0.0585*P*)^2^ + 0.4735*P*] where *P* = (*F*_o_^2^ + 2*F*_c_^2^)/3
*wR*(*F*^2^) = 0.132	(Δ/σ)_max_ < 0.001
*S* = 1.04	Δρ_max_ = 0.89 e Å^−^^3^
3865 reflections	Δρ_min_ = −0.56 e Å^−^^3^
309 parameters	

### Special details

**Table:**

Geometry. All e.s.d.'s (except the e.s.d. in the dihedral angle between two l.s. planes) are estimated using the full covariance matrix. The cell e.s.d.'s are taken into account individually in the estimation of e.s.d.'s in distances, angles and torsion angles; correlations between e.s.d.'s in cell parameters are only used when they are defined by crystal symmetry. An approximate (isotropic) treatment of cell e.s.d.'s is used for estimating e.s.d.'s involving l.s. planes.
Refinement. Refinement of *F*^2^ against ALL reflections. The weighted *R*-factor *wR* and goodness of fit *S* are based on *F*^2^, conventional *R*-factors *R* are based on *F*, with *F* set to zero for negative *F*^2^. The threshold expression of *F*^2^ > σ(*F*^2^) is used only for calculating *R*-factors(gt) *etc*. and is not relevant to the choice of reflections for refinement. *R*-factors based on *F*^2^ are statistically about twice as large as those based on *F*, and *R*- factors based on ALL data will be even larger.

### Fractional atomic coordinates and isotropic or equivalent isotropic displacement parameters (Å^2^)

**Table:**

	*x*	*y*	*z*	*U*_iso_*/*U*_eq_	
Fe1	0.05963 (6)	0.16467 (3)	0.33793 (2)	0.01446 (18)	
S1	0.05329 (11)	0.50871 (6)	0.35618 (5)	0.0211 (2)	
S2	0.05068 (11)	−0.17806 (6)	0.35648 (5)	0.0203 (2)	
O1	0.3101 (3)	0.16590 (15)	0.39010 (12)	0.0196 (5)	
H1A	0.3708	0.2145	0.3972	0.029*	
H1B	0.3752	0.1195	0.3998	0.029*	
O2	0.1789 (3)	0.16476 (14)	0.24007 (11)	0.0161 (5)	
H2A	0.2438	0.2119	0.2407	0.024*	
H2B	0.2417	0.1169	0.2393	0.024*	
O3	−0.0890 (3)	0.16644 (15)	0.41931 (12)	0.0203 (5)	
H3A	−0.1473	0.2146	0.4255	0.030*	
H3B	−0.1482	0.1196	0.4278	0.030*	
N1	0.3789 (4)	0.47023 (19)	0.06694 (14)	0.0183 (7)	
N2	0.2966 (4)	0.3219 (2)	0.06431 (16)	0.0258 (7)	
N3	0.4698 (4)	0.3241 (2)	0.08670 (14)	0.0202 (7)	
N4	0.3743 (4)	0.8658 (2)	0.05758 (14)	0.0174 (7)	
N5	0.2863 (4)	1.0131 (2)	0.05359 (15)	0.0224 (7)	
N6	0.4616 (4)	1.0122 (2)	0.07812 (14)	0.0209 (7)	
N7	0.5000	0.8633 (3)	0.2500	0.0154 (9)	
N8	0.4102 (4)	1.0106 (2)	0.24412 (14)	0.0214 (7)	
N9	0.5000	0.4665 (3)	0.2500	0.0145 (9)	
N10	0.4106 (4)	0.31863 (18)	0.24409 (14)	0.0168 (6)	
N11	0.0567 (4)	0.3124 (2)	0.33920 (14)	0.0183 (7)	
N12	0.0565 (4)	0.0182 (2)	0.34003 (14)	0.0196 (7)	
C1	0.2475 (5)	0.4088 (3)	0.05327 (19)	0.0261 (9)	
H1	0.1326	0.4268	0.0374	0.031*	
C2	0.5163 (5)	0.4124 (3)	0.08729 (17)	0.0216 (9)	
H2	0.6307	0.4339	0.1002	0.026*	
C3	0.3775 (4)	0.5706 (2)	0.06257 (17)	0.0169 (8)	
C4	0.2300 (5)	0.6193 (3)	0.03582 (18)	0.0198 (8)	
H4	0.1292	0.5851	0.0193	0.024*	
C5	0.2278 (5)	0.7155 (2)	0.03294 (18)	0.0187 (8)	
H5	0.1260	0.7478	0.0147	0.022*	
C6	0.3744 (4)	0.7656 (2)	0.05673 (17)	0.0158 (8)	
C7	0.5252 (4)	0.7171 (3)	0.08215 (17)	0.0194 (8)	
H7	0.6272	0.7513	0.0972	0.023*	
C8	0.5258 (4)	0.6211 (2)	0.08527 (17)	0.0170 (8)	
H8	0.6278	0.5887	0.1030	0.020*	
C9	0.2403 (5)	0.9256 (2)	0.04204 (18)	0.0212 (8)	
H9	0.1269	0.9063	0.0249	0.025*	
C10	0.5096 (5)	0.9249 (2)	0.07933 (17)	0.0201 (8)	
H10	0.6239	0.9043	0.0934	0.024*	
C11	0.3611 (5)	0.9233 (2)	0.24117 (18)	0.0207 (8)	
H11	0.2443	0.9034	0.2339	0.025*	
C12	0.5000	0.7624 (3)	0.2500	0.0139 (10)	
C13	0.3455 (4)	0.7139 (2)	0.23747 (16)	0.0151 (8)	
H13	0.2401	0.7475	0.2290	0.018*	
C14	0.3453 (4)	0.6164 (2)	0.23733 (17)	0.0178 (8)	
H14	0.2396	0.5830	0.2286	0.021*	
C15	0.5000	0.5672 (3)	0.2500	0.0161 (11)	
C16	0.3622 (5)	0.4059 (2)	0.24096 (18)	0.0199 (8)	
H16	0.2454	0.4256	0.2333	0.024*	
C17	0.0552 (4)	0.3932 (2)	0.34653 (17)	0.0155 (8)	
C18	0.0545 (4)	−0.0633 (2)	0.34701 (17)	0.0159 (8)	

### Atomic displacement parameters (Å^2^)

**Table:**

	*U*^11^	*U*^22^	*U*^33^	*U*^12^	*U*^13^	*U*^23^
Fe1	0.0105 (3)	0.0110 (3)	0.0222 (3)	0.0003 (2)	0.0035 (2)	−0.0001 (2)
S1	0.0128 (5)	0.0146 (5)	0.0355 (5)	0.0013 (4)	0.0016 (4)	−0.0022 (4)
S2	0.0134 (5)	0.0110 (5)	0.0363 (6)	−0.0011 (4)	0.0024 (4)	0.0023 (4)
O1	0.0137 (12)	0.0117 (12)	0.0329 (14)	0.0002 (10)	0.0011 (10)	0.0002 (10)
O2	0.0116 (11)	0.0094 (12)	0.0273 (13)	0.0001 (10)	0.0027 (10)	0.0005 (10)
O3	0.0154 (12)	0.0151 (13)	0.0318 (14)	−0.0017 (10)	0.0087 (10)	−0.0010 (10)
N1	0.0179 (16)	0.0148 (16)	0.0220 (16)	0.0038 (13)	0.0017 (13)	0.0006 (12)
N2	0.0175 (17)	0.0259 (19)	0.0356 (19)	−0.0038 (15)	0.0096 (14)	−0.0005 (15)
N3	0.0170 (16)	0.0218 (18)	0.0219 (16)	0.0009 (14)	0.0032 (13)	0.0026 (13)
N4	0.0153 (16)	0.0153 (15)	0.0220 (16)	−0.0004 (12)	0.0045 (13)	−0.0010 (12)
N5	0.0171 (16)	0.0194 (18)	0.0322 (18)	−0.0028 (14)	0.0088 (13)	−0.0003 (14)
N6	0.0216 (17)	0.0161 (17)	0.0254 (17)	−0.0034 (14)	0.0043 (13)	−0.0040 (13)
N7	0.010 (2)	0.009 (2)	0.027 (2)	0.000	0.0032 (17)	0.000
N8	0.0170 (15)	0.0242 (18)	0.0238 (16)	0.0008 (14)	0.0055 (13)	−0.0015 (14)
N9	0.012 (2)	0.015 (2)	0.017 (2)	0.000	0.0041 (16)	0.000
N10	0.0179 (15)	0.0117 (16)	0.0218 (16)	−0.0027 (12)	0.0062 (13)	−0.0014 (12)
N11	0.0131 (16)	0.0175 (18)	0.0251 (17)	0.0012 (12)	0.0056 (12)	−0.0001 (12)
N12	0.0148 (16)	0.0166 (18)	0.0276 (17)	−0.0017 (13)	0.0041 (13)	−0.0012 (13)
C1	0.016 (2)	0.026 (2)	0.037 (2)	0.0022 (17)	0.0070 (17)	0.0015 (17)
C2	0.018 (2)	0.025 (2)	0.022 (2)	0.0054 (16)	0.0028 (16)	0.0033 (16)
C3	0.0150 (19)	0.0174 (19)	0.0188 (19)	0.0012 (15)	0.0036 (15)	0.0013 (14)
C4	0.0099 (19)	0.023 (2)	0.026 (2)	−0.0004 (15)	0.0001 (15)	−0.0012 (16)
C5	0.0122 (19)	0.0158 (19)	0.028 (2)	0.0026 (15)	0.0010 (15)	0.0012 (15)
C6	0.0147 (19)	0.0156 (19)	0.0179 (18)	−0.0023 (14)	0.0055 (14)	−0.0029 (14)
C7	0.0107 (19)	0.026 (2)	0.0211 (19)	−0.0022 (15)	0.0005 (15)	−0.0027 (16)
C8	0.0112 (19)	0.021 (2)	0.0188 (19)	0.0020 (15)	0.0032 (15)	0.0015 (15)
C9	0.0134 (19)	0.020 (2)	0.031 (2)	−0.0017 (16)	0.0069 (16)	−0.0036 (16)
C10	0.017 (2)	0.019 (2)	0.025 (2)	−0.0038 (15)	0.0042 (16)	−0.0002 (15)
C11	0.018 (2)	0.020 (2)	0.025 (2)	−0.0030 (16)	0.0054 (16)	−0.0005 (15)
C12	0.014 (3)	0.013 (3)	0.015 (2)	0.000	0.005 (2)	0.000
C13	0.0118 (19)	0.0142 (19)	0.0195 (19)	0.0029 (14)	0.0034 (14)	0.0013 (14)
C14	0.0100 (19)	0.021 (2)	0.023 (2)	0.0008 (15)	0.0021 (15)	−0.0005 (15)
C15	0.017 (3)	0.015 (3)	0.016 (3)	0.000	0.004 (2)	0.000
C16	0.0164 (19)	0.021 (2)	0.022 (2)	−0.0039 (16)	0.0032 (15)	−0.0008 (15)
C17	0.0083 (18)	0.016 (2)	0.0226 (19)	−0.0017 (14)	0.0019 (14)	0.0054 (15)
C18	0.0075 (17)	0.022 (2)	0.0189 (19)	−0.0007 (15)	0.0021 (14)	−0.0040 (15)

### Geometric parameters (Å, º)

**Table:**

Fe1—N12	2.080 (3)	N8—N8^ii^	1.396 (6)
Fe1—N11	2.098 (3)	N9—C16^ii^	1.374 (4)
Fe1—O1	2.099 (2)	N9—C16	1.374 (4)
Fe1—O3	2.106 (2)	N9—C15	1.430 (6)
Fe1—O2	2.258 (2)	N10—C16	1.294 (4)
Fe1—O2^i^	2.271 (2)	N10—N10^ii^	1.390 (6)
S1—C17	1.652 (4)	N11—C17	1.156 (4)
S2—C18	1.641 (4)	N12—C18	1.166 (4)
O1—H1A	0.8400	C1—H1	0.9500
O1—H1B	0.8400	C2—H2	0.9500
O2—Fe1^i^	2.271 (2)	C3—C8	1.390 (5)
O2—H2A	0.8401	C3—C4	1.392 (5)
O2—H2B	0.8401	C4—C5	1.367 (5)
O3—H3A	0.8399	C4—H4	0.9500
O3—H3B	0.8400	C5—C6	1.382 (5)
N1—C1	1.350 (4)	C5—H5	0.9500
N1—C2	1.373 (4)	C6—C7	1.405 (5)
N1—C3	1.428 (4)	C7—C8	1.364 (5)
N2—C1	1.303 (4)	C7—H7	0.9500
N2—N3	1.373 (4)	C8—H8	0.9500
N3—C2	1.304 (4)	C9—H9	0.9500
N4—C9	1.355 (4)	C10—H10	0.9500
N4—C10	1.378 (4)	C11—H11	0.9500
N4—C6	1.423 (4)	C12—C13	1.387 (4)
N5—C9	1.306 (4)	C12—C13^ii^	1.387 (4)
N5—N6	1.397 (4)	C13—C14	1.385 (5)
N6—C10	1.295 (4)	C13—H13	0.9500
N7—C11^ii^	1.376 (4)	C14—C15	1.393 (4)
N7—C11	1.376 (4)	C14—H14	0.9500
N7—C12	1.432 (6)	C15—C14^ii^	1.393 (4)
N8—C11	1.296 (4)	C16—H16	0.9500
			
N12—Fe1—N11	177.62 (11)	N1—C1—H1	123.9
N12—Fe1—O1	90.63 (10)	N3—C2—N1	111.4 (3)
N11—Fe1—O1	89.82 (10)	N3—C2—H2	124.3
N12—Fe1—O3	89.29 (10)	N1—C2—H2	124.3
N11—Fe1—O3	88.33 (10)	C8—C3—C4	119.1 (3)
O1—Fe1—O3	101.16 (9)	C8—C3—N1	119.7 (3)
N12—Fe1—O2	91.43 (9)	C4—C3—N1	121.2 (3)
N11—Fe1—O2	90.93 (9)	C5—C4—C3	121.2 (3)
O1—Fe1—O2	87.86 (9)	C5—C4—H4	119.4
O3—Fe1—O2	170.94 (8)	C3—C4—H4	119.4
N12—Fe1—O2^i^	90.20 (9)	C4—C5—C6	119.6 (3)
N11—Fe1—O2^i^	89.90 (9)	C4—C5—H5	120.2
O1—Fe1—O2^i^	166.73 (9)	C6—C5—H5	120.2
O3—Fe1—O2^i^	92.09 (9)	C5—C6—C7	119.7 (3)
O2—Fe1—O2^i^	78.88 (9)	C5—C6—N4	121.1 (3)
Fe1—O1—H1A	124.3	C7—C6—N4	119.1 (3)
Fe1—O1—H1B	127.5	C8—C7—C6	120.2 (3)
H1A—O1—H1B	107.0	C8—C7—H7	119.9
Fe1—O2—Fe1^i^	101.12 (9)	C6—C7—H7	119.9
Fe1—O2—H2A	107.6	C7—C8—C3	120.2 (3)
Fe1^i^—O2—H2A	117.0	C7—C8—H8	119.9
Fe1—O2—H2B	108.7	C3—C8—H8	119.9
Fe1^i^—O2—H2B	114.9	N5—C9—N4	111.5 (3)
H2A—O2—H2B	106.9	N5—C9—H9	124.2
Fe1—O3—H3A	119.0	N4—C9—H9	124.2
Fe1—O3—H3B	121.1	N6—C10—N4	111.6 (3)
H3A—O3—H3B	107.0	N6—C10—H10	124.2
C1—N1—C2	102.8 (3)	N4—C10—H10	124.2
C1—N1—C3	129.3 (3)	N8—C11—N7	111.2 (3)
C2—N1—C3	127.9 (3)	N8—C11—H11	124.4
C1—N2—N3	106.9 (3)	N7—C11—H11	124.4
C2—N3—N2	106.8 (3)	C13—C12—C13^ii^	120.5 (4)
C9—N4—C10	103.4 (3)	C13—C12—N7	119.8 (2)
C9—N4—C6	128.7 (3)	C13^ii^—C12—N7	119.8 (2)
C10—N4—C6	127.7 (3)	C14—C13—C12	119.9 (3)
C9—N5—N6	106.9 (3)	C14—C13—H13	120.1
C10—N6—N5	106.6 (3)	C12—C13—H13	120.1
C11^ii^—N7—C11	103.4 (4)	C13—C14—C15	120.0 (4)
C11^ii^—N7—C12	128.3 (2)	C13—C14—H14	120.0
C11—N7—C12	128.3 (2)	C15—C14—H14	120.0
C11—N8—N8^ii^	107.1 (2)	C14—C15—C14^ii^	119.9 (5)
C16^ii^—N9—C16	102.4 (4)	C14—C15—N9	120.1 (2)
C16^ii^—N9—C15	128.8 (2)	C14^ii^—C15—N9	120.1 (2)
C16—N9—C15	128.8 (2)	N10—C16—N9	111.9 (3)
C16—N10—N10^ii^	106.9 (2)	N10—C16—H16	124.0
C17—N11—Fe1	173.5 (3)	N9—C16—H16	124.0
C18—N12—Fe1	174.3 (3)	N11—C17—S1	179.4 (3)
N2—C1—N1	112.2 (3)	N12—C18—S2	179.7 (4)
N2—C1—H1	123.9		
			
N12—Fe1—O2—Fe1^i^	89.86 (10)	C9—N4—C6—C7	−172.9 (3)
N11—Fe1—O2—Fe1^i^	−89.78 (10)	C10—N4—C6—C7	1.9 (5)
O1—Fe1—O2—Fe1^i^	−179.56 (9)	C5—C6—C7—C8	−2.0 (5)
O3—Fe1—O2—Fe1^i^	−4.6 (6)	N4—C6—C7—C8	176.5 (3)
O2^i^—Fe1—O2—Fe1^i^	−0.06 (11)	C6—C7—C8—C3	0.7 (5)
C1—N2—N3—C2	−0.6 (4)	C4—C3—C8—C7	0.9 (5)
C9—N5—N6—C10	0.4 (4)	N1—C3—C8—C7	−179.1 (3)
N12—Fe1—N11—C17	33 (4)	N6—N5—C9—N4	0.2 (4)
O1—Fe1—N11—C17	−67 (2)	C10—N4—C9—N5	−0.7 (4)
O3—Fe1—N11—C17	34 (2)	C6—N4—C9—N5	175.1 (3)
O2—Fe1—N11—C17	−155 (2)	N5—N6—C10—N4	−0.8 (4)
O2^i^—Fe1—N11—C17	126 (2)	C9—N4—C10—N6	0.9 (4)
N11—Fe1—N12—C18	−32 (5)	C6—N4—C10—N6	−174.9 (3)
O1—Fe1—N12—C18	69 (3)	N8^ii^—N8—C11—N7	−0.4 (4)
O3—Fe1—N12—C18	−32 (3)	C11^ii^—N7—C11—N8	0.16 (17)
O2—Fe1—N12—C18	157 (3)	C12—N7—C11—N8	−179.84 (17)
O2^i^—Fe1—N12—C18	−124 (3)	C11^ii^—N7—C12—C13	−177.4 (2)
N3—N2—C1—N1	0.1 (4)	C11—N7—C12—C13	2.6 (2)
C2—N1—C1—N2	0.4 (4)	C11^ii^—N7—C12—C13^ii^	2.6 (2)
C3—N1—C1—N2	−178.8 (3)	C11—N7—C12—C13^ii^	−177.4 (2)
N2—N3—C2—N1	1.0 (4)	C13^ii^—C12—C13—C14	−0.1 (2)
C1—N1—C2—N3	−0.9 (4)	N7—C12—C13—C14	179.9 (2)
C3—N1—C2—N3	178.4 (3)	C12—C13—C14—C15	0.2 (4)
C1—N1—C3—C8	174.0 (3)	C13—C14—C15—C14^ii^	−0.1 (2)
C2—N1—C3—C8	−5.0 (5)	C13—C14—C15—N9	179.9 (2)
C1—N1—C3—C4	−6.1 (6)	C16^ii^—N9—C15—C14	177.6 (3)
C2—N1—C3—C4	174.9 (3)	C16—N9—C15—C14	−2.4 (3)
C8—C3—C4—C5	−1.4 (5)	C16^ii^—N9—C15—C14^ii^	−2.4 (3)
N1—C3—C4—C5	178.6 (3)	C16—N9—C15—C14^ii^	177.6 (3)
C3—C4—C5—C6	0.2 (5)	N10^ii^—N10—C16—N9	0.1 (4)
C4—C5—C6—C7	1.5 (5)	C16^ii^—N9—C16—N10	−0.04 (17)
C4—C5—C6—N4	−176.9 (3)	C15—N9—C16—N10	179.96 (17)
C9—N4—C6—C5	5.5 (5)	Fe1—N11—C17—S1	176 (100)
C10—N4—C6—C5	−179.7 (3)	Fe1—N12—C18—S2	126 (70)

Symmetry codes: (i) −*x*, *y*, −*z*+1/2; (ii) −*x*+1, *y*, −*z*+1/2.

### Hydrogen-bond geometry (Å, º)

**Table:**

*D*—H···*A*	*D*—H	H···*A*	*D*···*A*	*D*—H···*A*
O1—H1*A*···N3^ii^	0.84	2.00	2.833 (4)	176
O1—H1*B*···N6^iii^	0.84	2.00	2.841 (4)	178
O2—H2*A*···N10	0.84	2.00	2.834 (3)	176
O2—H2*B*···N8^iv^	0.84	2.00	2.836 (4)	174
O3—H3*A*···N2^i^	0.84	1.95	2.784 (4)	176
O3—H3*B*···N5^v^	0.84	1.92	2.761 (4)	179

Symmetry codes: (i) −*x*, *y*, −*z*+1/2; (ii) −*x*+1, *y*, −*z*+1/2; (iii) −*x*+1, *y*−1, −*z*+1/2; (iv) *x*, *y*−1, *z*; (v) −*x*, *y*−1, −*z*+1/2.
